# Semantically enabling a genome-wide association study database

**DOI:** 10.1186/2041-1480-3-9

**Published:** 2012-12-17

**Authors:** Tim Beck, Robert C Free, Gudmundur A Thorisson, Anthony J Brookes

**Affiliations:** 1 Department of Genetics, University of Leicester, University Road, Leicester, UK

**Keywords:** Ontology, Phenotype, GWAS, RDF

## Abstract

**Background:**

The amount of data generated from genome-wide association studies (GWAS) has grown rapidly, but considerations for GWAS phenotype data reuse and interchange have not kept pace. This impacts on the work of GWAS Central – a free and open access resource for the advanced querying and comparison of summary-level genetic association data. The benefits of employing ontologies for standardising and structuring data are widely accepted. The complex spectrum of observed human phenotypes (and traits), and the requirement for cross-species phenotype comparisons, calls for reflection on the most appropriate solution for the organisation of human phenotype data. The Semantic Web provides standards for the possibility of further integration of GWAS data and the ability to contribute to the web of Linked Data.

**Results:**

A pragmatic consideration when applying phenotype ontologies to GWAS data is the ability to retrieve all data, at the most granular level possible, from querying a single ontology graph. We found the Medical Subject Headings (MeSH) terminology suitable for describing all traits (diseases and medical signs and symptoms) at various levels of granularity and the Human Phenotype Ontology (HPO) most suitable for describing phenotypic abnormalities (medical signs and symptoms) at the most granular level. Diseases within MeSH are mapped to HPO to infer the phenotypic abnormalities associated with diseases. Building on the rich semantic phenotype annotation layer, we are able to make cross-species phenotype comparisons and publish a core subset of GWAS data as RDF nanopublications.

**Conclusions:**

We present a methodology for applying phenotype annotations to a comprehensive genome-wide association dataset and for ensuring compatibility with the Semantic Web. The annotations are used to assist with cross-species genotype and phenotype comparisons. However, further processing and deconstructions of terms may be required to facilitate automatic phenotype comparisons. The provision of GWAS nanopublications enables a new dimension for exploring GWAS data, by way of intrinsic links to related data resources within the Linked Data web. The value of such annotation and integration will grow as more biomedical resources adopt the standards of the Semantic Web.

## Background

**Figure 1:**
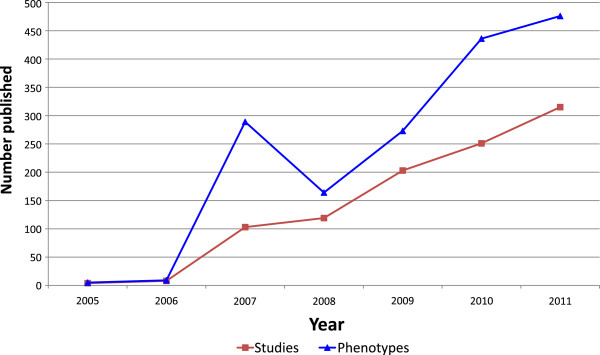
**The number of GWAS published and the phenotypes reported each year.** Since 2005 there has been year-on-year growth in the number of published GWAS. The number of phenotypes reported each year has consistently remained higher than the number of studies since 2006, indicating a preference to report individual phenotypic components of a disease. Data from GWAS Central.

In recent years the amount of data generated from genome-wide association studies (GWAS) has increased rapidly. However, the formal representation and description of those data, especially with regards to phenotype, has lagged behind. The publication of the first successful GWAS in 2005 heralded the start of an exciting new era of genetic research that would go on to contribute substantially to our understanding of disease mechanisms, such as the discovery of novel genes linked to Crohn’s disease and age-related macular degeneration [1]. By March 2008 over one hundred GWAS had been published, and that number was growing nearly exponentially [2]. The rapid rate of growth has been sustained, and so by the start of 2012, over one thousand published GWAS papers are available in the literature (Figure 1; red line).

The database resource GWAS Central http://www.gwascentral.org (established in 2007, then named HGVbaseG2P [3]) is a comprehensive central collection of genetic association data with a focus on advanced tools to integrate, search and compare summary-level data sets. GWAS Central is a core component of the GEN2PHEN project http://www.gen2phen.org, which aims to unify human and model organism genetic variation databases. The modular architecture of GWAS Central allows the infrastructure to be extended for use with different types of data, and it is anticipated that through future support from the BioSHaRE project http://www.bioshare.eu, GWAS Central will be extended to integrate exome and next-generation sequencing data.

Currently, GWAS Central collates data from a range of sources, including the published literature, collaborating databases such as the NHGRI GWAS Catalog [4], and direct submissions from collaborating investigators. A given study represented in GWAS Central may investigate the genetic association to a single phenotype, or a range of phenotypes, associated with a disease of interest. In the case of multiple phenotypes, “sub-studies” will be reported as separate experiments. For example, a single GWAS may identify common genetic variation altering the risk to type 2 diabetes susceptibility, and so report the results from single or multiple experiments investigating related traits such as fasting plasma glucose levels, insulin sensitivity index, insulin response or findings from a glucose tolerance test. GWAS Central captures this distinction and reports the individual phenotype tested as well as the disease of interest.

GWAS Central currently holds 1664 reported phenotypes (Figure 1; blue line). Identical phenotypes may be described differently between studies due to inconsistencies associated with variations in terminology use and in editorial style of authors when describing the phenotypes. A pragmatic solution was required to allow harmonisation of the GWAS phenotype descriptions to facilitate consistent querying within GWAS Central, and to ensure that the phenotype data can be accessed and understood using a semantic standard to allow data integration.

### Ontologies for GWAS information

The benefits of ontologies in resolving ambiguity associated with divergent and “free-text” nomenclature are well documented [5]. The issues surrounding the reusability of phenotype descriptions within GWAS Central are typical of problems tackled by groups working on the controlled vocabulary of other model organisms, for example yeast [6], worm [7] and mouse [8]. In these cases, either new phenotype ontologies were built or existing ontologies were applied within a meaningful annotation framework.

The Open Biological and Biomedical Ontologies (OBO) Foundry is an initiative involving the developers of life-science ontologies and is tasked with setting principles for ontology development. OBO’s goal is to coordinate development of a collection of orthogonal interoperable biomedical ontologies to support data integration [9]. The application of two OBO Foundry principles in particular suggest that the development of a new ontology to capture human phenotype data derived from GWAS would not be in the community’s best interest. These principles assert that new ontologies must be, firstly, orthogonal to other ontologies already lodged within OBO, and secondly, contain a plurality of mutually independent users [10].

One candidate OBO Foundry ontology in name alone - the Human Phenotype Ontology (HPO) [11] - indicates immediate overlap with our domain of interest (GWAS phenotypes). Further human phenotype-related ontologies are also available from the National Center for Biomedical Ontology (NCBO) BioPortal [12], for example Medical Subject Headings (MeSH) [13] and the International Classification of Diseases (ICD) [14]. Despite OBO Foundry efforts in promoting the creation of orthogonal ontologies, there is still a high rate of term reuse, with a recent study reporting 96% of Foundry candidate ontologies using terms from other ontologies [15]. The prevalence of term reuse and redundancy between ontologies leaves potential users asking the obvious question “which ontology do I use?”.

The ambiguity in arriving at an obvious candidate ontology can have a devastating effect on system interoperability and data interchange. We believe the development of a dedicated GWAS phenotype ontology would compound that problem. Additionally, since 2007 when HGVbaseG2P was established, there has been no call for a dedicated GWAS phenotype ontology from other quarters, so also failing the “plurality of users” principle. Consideration of these factors led us to favour an approach that involves the application of existing ontologies within the GWAS Central data model.

### Phenotypes, traits, medical signs and symptoms

In the context of the genetic analysis of human disease, and thus GWAS, the term ‘phenotype’ is used to define an aggregated set of medically and semantically distinct concepts. Traits and phenotypes are often considered synonymous, however they are distinct domains within Ontology. A trait is a heritable, measurable or identifiable characteristic of an organism such as systolic blood pressure. Phenotype is a scalar trait [16], essentially a trait with a value, such as increased systolic blood pressure. GWAS typically report findings in relation to traits, for example “Genome-wide association study identifies eight loci associated with blood pressure” [17]. Furthermore, human disease is a complex collection of phenotypic observations and pathological processes [18]. The diagnosis of a disease depends upon identifying a set of phenotypes, which can be either medical signs or symptoms. A medical sign is an objective indication of a medical characteristic that can be detected by a healthcare professional such as blood pressure. A symptom is a subjective observation of the patient that their feeling or function has departed from the ‘normal’ such as experiencing pain. GWAS report genetic associations to diseases, for example, “Candidate single-nucleotide polymorphisms from a genomewide association study of Alzheimer disease” [19], and also medical signs and symptoms such as “Genome-wide association study of acute post-surgical pain in humans” [20].

During the course of this study, which sets out to implement a strategy for logically describing and distributing GWAS observations contained within the GWAS Central database resource to support GWAS data comparison, we examine these differing granularities of phenotypes (or traits). Nonetheless, in order to aid readability throughout this manuscript we use the term ‘phenotype’, unless otherwise stated, with the same all-encompassing meaning assumed by the biologist: namely, the observable characteristics resulting from the expression of genes and the influence of environmental factors.

### Cross-species phenotype analysis for validating GWAS

A striking advantage of binding human GWAS phenotypes to an ontology is the ability to extend automatic cross-species analyses of phenotype and genotype information with comparative, suitably annotated, datasets. The laboratory mouse is a central model organism for the analysis of mammalian development, physiological and disease processes [21]. It is therefore understandable that the mouse has been suggested as an ideal model for the functional validation of GWAS results [22].

A range of resources are available for the querying of mouse genotype-phenotype associations, such as: the Mouse Genome Database (MGD) which contains data loaded from other databases, from direct submissions, and from the published literature [23]; EuroPhenome, a repository for high-throughput mouse phenotyping data [24]; advanced semantics infrastructure involving development of a species-neutral anatomy ontology [25]; and finally a unified specification for representing phenotypes across species as *entities* and *qualities* (EQ) [26] that has been proposed to enable the linking of mouse phenotypes to human diseases and phenotypes for comparative genome-phenome analysis [27].

A major bottleneck in implementing high-throughput phenomic comparisons leveraging the above resources is the absence of a well annotated, controlled and accessible human disease genotype-phenotype dataset, and the necessary tools to access it.

### Linked GWAS data and the Semantic Web

The Semantic Web builds upon the Resource Description Framework (RDF) and related standards to give meaning to unstructured documents on the web to allow data to be understood, shared and reused. The term “Linked Data” is commonly used to refer to a specific approach to connecting data, information and knowledge on the Semantic Web that was not previously linked [28]. These technologies and approaches have in recent years been slowly but surely infiltrating the life sciences domain to tackle diverse problems. A notable recent development is the Semantic Automated Discovery and Integration framework (SADI) [29], a set of conventions for using Semantic Web standards to automate the construction of analytical workflows.

In the field of disease genetics, applications of Semantic Web technologies range from publishing information held in curated locus-specific databases as Linked Data [30], to text-mining the published scientific literature for mutations found to affect protein structure and subsequently making methods and data accessible via the SADI framework [31,32]. To our knowledge, this has not yet been done with GWAS data in a comprehensive fashion. In relation to the Linked Data approach specifically, enhancement of GWAS datasets (such as those made available via GWAS Central) with phenotype annotations published in Semantic Web compatible formats has the potential to facilitate integration with other, related, Linked Data resources, such as genes, proteins, diseases and publications [33,34].

The complexity of GWAS data sets and associated metadata led us to adopt so-called “nanopublications” [35]; a recently developed framework for publishing one or more scientific assertions as Linked Data, wrapped into self-contained “bundles” which also contain the contextual information necessary for the interpretation of the assertion, as well as provenance, attribution and other key metadata. The nanopublishing approach has already been used to publish locus-specific data [36] and other biological datasets [37]. Ultimately, by making a comprehensive GWAS dataset available as nanopublications we aim to provide a rich addition to the web of Linked Data, while also allowing researchers who contribute to primary GWAS publications to be properly attributed. This latter feature of nanopublications is a compelling reason for their use, particularly with the recent drive towards publishing data and metadata and creating incentives for researchers to share their data [38].

## Results

### Analysis of ontologies for describing GWAS phenotypes

Several ontologies available from the NCBO BioPortal could be used to annotate part or all of the phenotypes described by GWAS. Some of the most relevant ones are either members of the Unified Medical Language System (UMLS) BioPortal grouping (for example, MeSH, ICD10 and SNOMED CT [39]) or categorised by BioPortal as being related to ‘Phenotype’ (for example, HPO). We attempted to objectively identify which ontology would be most suitable for the purpose of defining GWAS phenotypes.

To this end, we defined *ontology suitability* as the ability to capture the maximum number of phenotypes at the level of granularity at which they are described. Our ambition to find a single ontology capable of describing the broad spectrum of GWAS phenotypes was pragmatically driven by a requirement to have a single ontology to query the entire database against. If we were to query against the complete ontology graph we would require all phenotypes to be returned. Therefore, during this comparative study we would consider an ontology more suitable if it could describe (either by concept or by synonym) the condition “Fuchs endothelial dystrophy” compared to the more general “corneal disease” or, more generally still, the term “eye disease”.

Since the majority of the ‘phenotype’ descriptions in GWAS Central are in fact trait descriptions (using the definition above) we assessed the suitability of HPO, ICD10, MeSH, SNOMED CT and also the Human Disease Ontology (DO) [40] for describing GWAS traits. The results from automatic exact and partial term mapping (see Methods) showed SNOMED CT and MeSH to be most suitable for mapping to the 1046 unique descriptions of GWAS traits (Table 1). Both could be mapped directly, after text normalisation (see Methods), to just over 20% of the traits exactly (MeSH 20.4% and SNOMED CT 21%). This compared with exactly mapping 10.8% of the traits with DO, 7% with HPO and 3.7% with ICD10.

**Table 1:** Results from the automatic mapping of GWAS phenotypes to relevant human-related vocabularies in BioPortal

**Vocabulary**	**Number of mappings**		**Coverage of GWAS Central phenotypes**		**Number of unique mappings**		**Unique coverage of the total automatic mappings made**	
	**Exact**	**Partial**	**Exact**	**Partial**	**Exact**	**Partial**	**Exact**	**Partial**
Human Disease Ontology (DO)	113	146	10.8%	14.0%	3	1	0.9%	0.2%
Human Phenotype Ontology (HPO)	73	140	7.0%	13.4%	16	30	4.8%	6.9%
International Classification of Diseases (ICD10)	39	81	3.7%	7.7%	0	0	0%	0%
Medical Subject Headings (MeSH)	213	256	20.4%	24.5%	51	29	15.4%	6.7%
Systematized Nomenclature of Medicine Clinical Terms (SNOMED CT)	220	291	21.0%	27.8%	33	53	9.9%	12.2%

The numbers of mappings achieved for both the exact and partial searches are shown along with the percentage of the total number of GWAS Central phenotypes this relates to. A total of 1046 distinct trait descriptions were assessed and 332 were exactly mapped and 434 partially mapped to terms in one or more of the vocabularies. The number of cases where the vocabulary in question is the only vocabulary to map to a particular trait description is given in the “Number of unique mappings” column, followed by the percentage of the total number of automatic annotations this represents.

The decision to adopt MeSH as the “backbone” for GWAS phenotype annotations in GWAS Central was taken due to MeSH being more familiar to biologists compared to the clinically focussed SNOMED CT. MeSH is used by the U.S. National Library of Medicine’s MEDLINE database to index abstracts and is searchable in PubMed [41]. By contrast, there are relatively few research-related implementations of SNOMED CT. Additionally, SNOMED CT is more difficult to navigate and manage compared to MeSH, with SNOMED CT containing just under 400,000 classes compared to just under 230,000 in MeSH (figures taken from BioPortal).

In addition, we assessed the novel mappings achieved by each vocabulary (Table 1). Novel mappings occurred when a free-text phenotype description mapped to a term in a single ontology. During the exact mapping process, MeSH uniquely contributed 15.4% of the total 332 exactly mapped terms, followed by SNOMED CT (9.9%) and HPO (4.8%). However, during the partial mapping SNOMED CT uniquely contributed 12.2% of the total 434 partially mapped terms, followed by HPO (6.9%) and MeSH (6.7%). Inspection of the mapping results showed that by switching from exact mapping to partial mapping, a free-text phenotype description such as “forced expiratory volume” that had previously uniquely mapped to the MeSH Descriptor “Forced Expiratory Volume”, could now map to a SNOMED CT term “Normal forced expiratory volume”. Similarly, the free-text phenotype description “ventricular conduction” that could not map to any of the terminologies during the exact mapping could uniquely map to the SNOMED CT term “Ventricular conduction pattern” during the partial mapping. Since HPO made the second highest unique contribution in the partial mappings we assessed the benefits HPO could make in the annotation of GWAS phenotypes.

The HPO is an ontology of phenotypic abnormalities that was developed in order to provide a standardised basis for computational analysis of human disease manifestations [42]. The results from our ontology suitability analysis indicated that HPO would facilitate unique mapping of 30 GWAS phenotype descriptions during the partial mapping process. Manual inspection of these terms showed they were terms describing medical signs and symptoms, rather than disease names that have high coverage in the other ontologies investigated. For example, HPO can uniquely describe “Coronary artery calcification” (term identifier HP:0001717) rather than the disease for which this can be a clinical manifestation such as in “Gaucher Disease” (MeSH Descriptor identifier D005776).

The performance of HPO in mapping to GWAS traits increased from 7% for exact mappings to 13.4% for partial mappings (Table 1). Since HPO is an ontology of phenotypic abnormalities it contains many terms where the string “Abnormal” or similar precedes the trait. During the partial mapping, traits such as “number of teeth” mapped to partially related HPO terms such as “Abnormal number of teeth”, hence the improved performance of HPO in making unique term contributions during the partial mappings.

Not every medical sign and symptom in the GWAS Central phenotype description list could be mapped to HPO, due to either lack of an appropriate term or lack of a synonym. However, the HPO group seeks community engagement and there is a protocol in place for users to submit required terms for inclusion via the HPO term tracker [43]. Regular updates of the central ontology file ensure the changes are disseminated in a timely manner. In addition, subsets of terms from HPO are undergoing deconstruction into EQ descriptions [44], thus facilitating the use of HPO in cross-species comparisons. These factors made HPO a candidate for the annotation of individual phenotypic abnormalities (medical signs and symptoms) within GWAS Central.

The relatively low coverage overall achieved through automatic term mapping suggests that human decision-making is required during the process of phenotype curation, in order to ensure the biological meaning is preserved during the selection of alternative but appropriate, lexically distinct, concepts.

### Describing phenotypes using MeSH and HPO

MeSH is structured into a hierarchy of Descriptors (or Headings) under which Terms that are strictly synonymous with each other are grouped in a Concept category. The Descriptor/Concept/Term structure is adopted within GWAS Central. Each GWAS reported in GWAS Central undergoes a phenotype annotation process (see Methods). During the annotation process the original full-text published report of the GWAS is accessed via PubMed (or via communications with collaborating groups e.g. pre-publication reports) and all phenotypes for each experiment are manually curated with a MeSH Descriptor by a small team of postdoctoral experts to ensure a high level of quality and consistency.

Where possible, a Descriptor is assigned which is described by a Term that matches the phenotype under consideration exactly. Where an exact match cannot be found then the closest match is sought, usually by selecting the parent Descriptor in the hierarchy, from where the curator would expect the exact Descriptor to exist. For example, the phenotype “sporadic amyotrophic lateral sclerosis” would be annotated with the MeSH Descriptor “Amyotrophic Lateral Sclerosis”. If a published report has been indexed for MEDLINE, this indicates that subject analysts at the United States National Library of Medicine have examined the article and assigned the most specific MeSH terms applicable to the article [41]. In these cases the GWAS Central curators will consider any phenotype-related MEDLINE MeSH Descriptors for use alongside any additional appropriate MeSH Descriptors.

Phenotypes in GWAS Central are annotated at the level of individual experiments. This is in contrast to the MEDLINE MeSH annotations made at the level of the whole publication, which identify phenotypes that are mentioned somewhere in the journal article. GWAS Central curators are required to ensure that the correct phenotypes are associated with the correct experiments, which in turn are associated with the correct analysis methods, analysis and sample panels, and genetic marker datasets as defined by the GWAS Central data model (definitions of these concepts are available from the GWAS Central glossary: http://www.gwascentral.org/info/reference/definitions-and-glossary).

MEDLINE indexing is not available for all articles at the time of inclusion in GWAS Central. Citations supplied by publishers are not indexed and are identified by the citation status tag *[PubMed – as supplied by publisher]*, for example, the GWAS reported in the article by Paus *et al.* (2011) with a PubMed ID of 22156575 http://www.ncbi.nlm.nih.gov/pubmed/22156575. There can also be a delay from a GWAS report being made available in PubMed to it being indexed for MEDLINE, during which time the citation is assigned the status tag *[PubMed – in progress]*[42]. Since GWAS Central is frequently updated to ensure it contains the very latest studies, it is usual for the most recent reports not to contain MEDLINE MeSH annotations at the time of import.

The GWAS Central interface allows phenotypes to be retrieved via browsing the hierarchy of Descriptors (only Descriptors which are used in annotations are rendered) or by searching for Terms using an auto-suggest text field.

**Figure 2:**
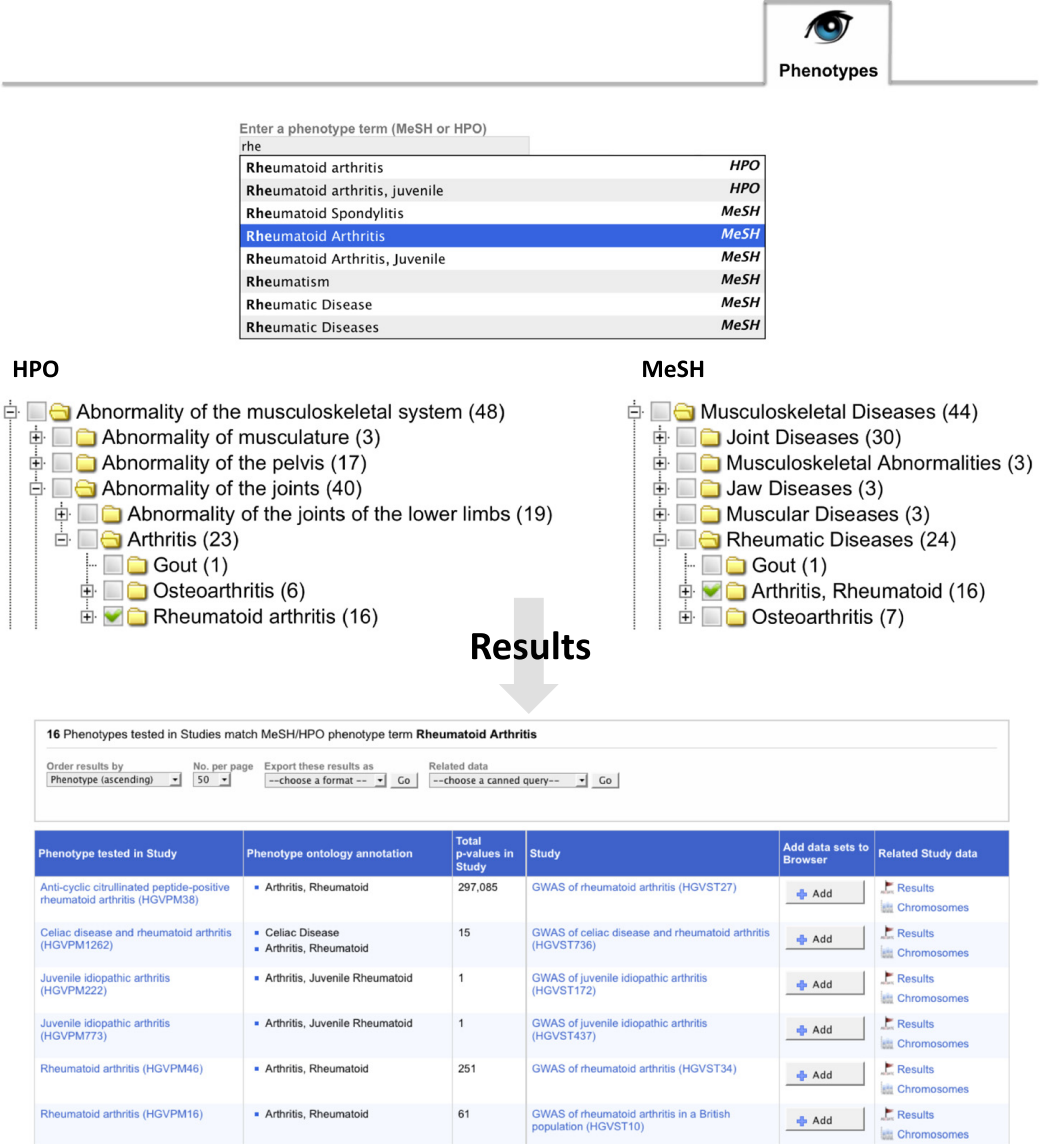
**Querying GWAS Central against phenotype ontology annotations.** A query for “rheumatoid arthritis” can be made by browsing either the MeSH or HPO hierarchy and selecting the appropriate term, or by using the auto-suggest text field. Only MeSH Descriptors or HPO terms used in annotations are displayed in the hierarchies. Only MeSH Terms or HPO terms and synonyms used in annotations are presented as suggested queries. The bracketed numbers after terms in the hierarchies represent the number of unique experiments annotated to that term. The first six hits of a total results list of sixteen experiments are shown.

In cases where a phenotype can be annotated to a greater resolution using HPO then this is done. In addition, a process of ontology mapping automatically annotates phenotypes to the corresponding HPO term from the original manually assigned MeSH annotation (see Methods). As with MeSH annotations, a HPO hierarchy containing only terms annotated to phenotypes can be browsed from the GWAS Central interface, and terms and synonyms can be queried using an auto-suggest text field (Figure 2).

### Inferring phenotypes for disease using HPO to OMIM mappings

The HPO defines the individual phenotypic abnormalities associated with a disease, rather than the disease itself. Therefore, when a disease name, such as “Creutzfeldt-Jakob Syndrome”, is used to describe a GWAS phenotype then a single HPO term representing the disease will not exist. Instead, HPO can be used to define the medical signs and symptoms associated with the disease. The HPO was originally constructed using data from the Online Mendelian Inheritance in Man (OMIM) database [45], and now provides comprehensive annotations of clinical phenotypes for OMIM diseases [11]. These HPO-to-OMIM mappings are implemented alongside OMIM-to-MeSH term mappings in GWAS Central to provide automatically inferred clinical manifestations described by HPO for the originally assigned disease annotation described by MeSH. These phenotypes are “inferred” since they may or may not be present, or present in differing severities, in the GWAS participants contributing to a study. While all participants for a study share the characteristic of having been diagnosed with the disease, it is not possible to determine from the GWAS report which medical signs or symptoms contributed to the diagnosis. The inferred HPO phenotypes indicate which clinical manifestations could have contributed to the diagnosis.

**Figure 3:**
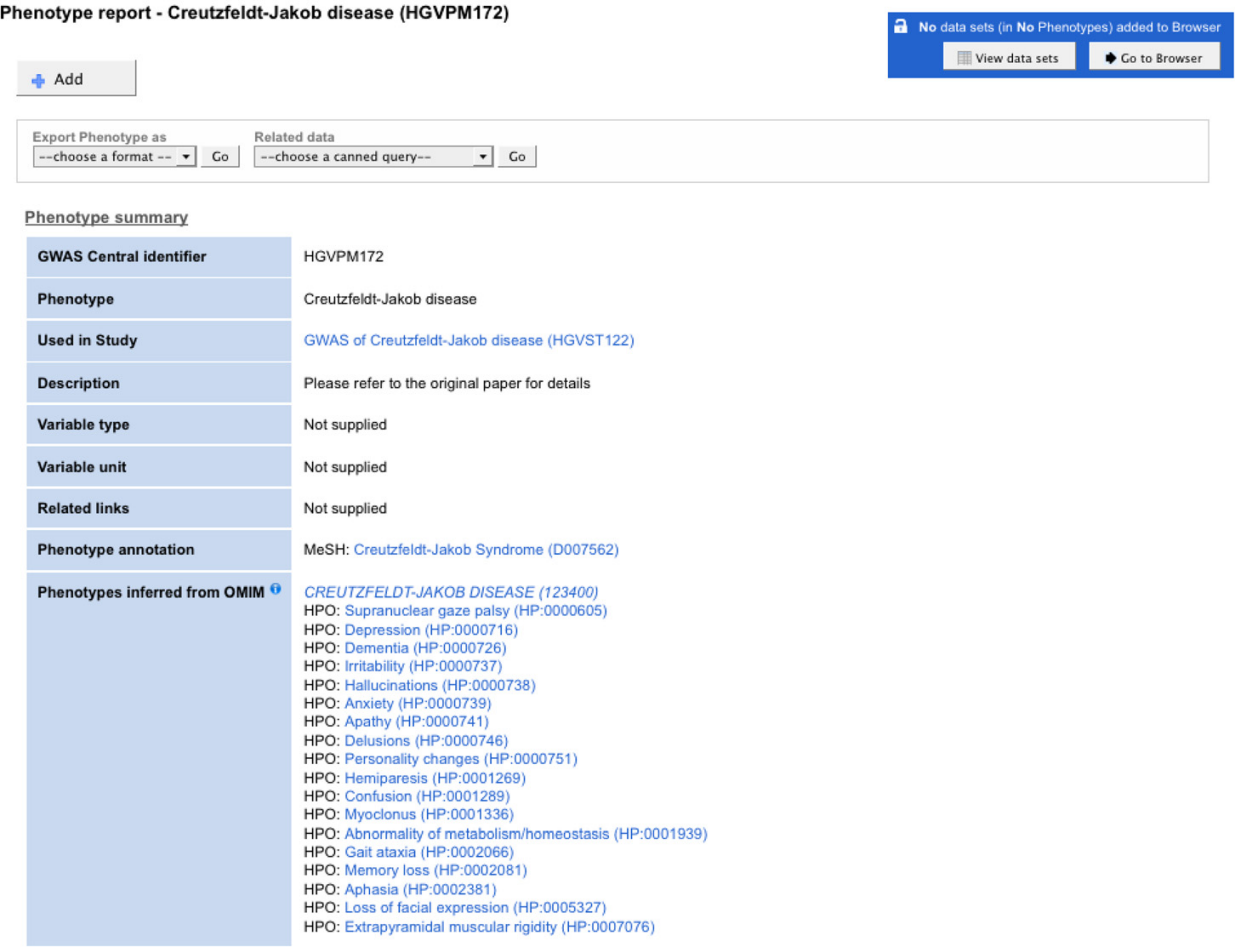
**Inferred phenotypes from OMIM as displayed in a GWAS Central “Phenotype Report”.** The phenotypic abnormalities associated with Creutzfeldt-Jakob Syndrome are listed under the OMIM term they are mapped to. A single MeSH disease Descriptor is associated with this GWAS experiment and the mappings are implemented ‘under the hood’ to provide clickable links to the mapped OMIM and HPO terms. Screenshot taken of http://www.gwascentral.org/phenotype/HGVPM172 .

A search in GWAS Central that returns a phenotype report annotated to MeSH disease Descriptor “Creutzfeldt-Jakob Syndrome” will display the mapping to the OMIM “Creutzfeldt-Jakob Disease” entry and the HPO-to-OMIM derived phenotypic abnormalities for the disease, which include “Confusion” and “Loss of facial expression”, amongst others (Figure 3).

**Figure 4:**
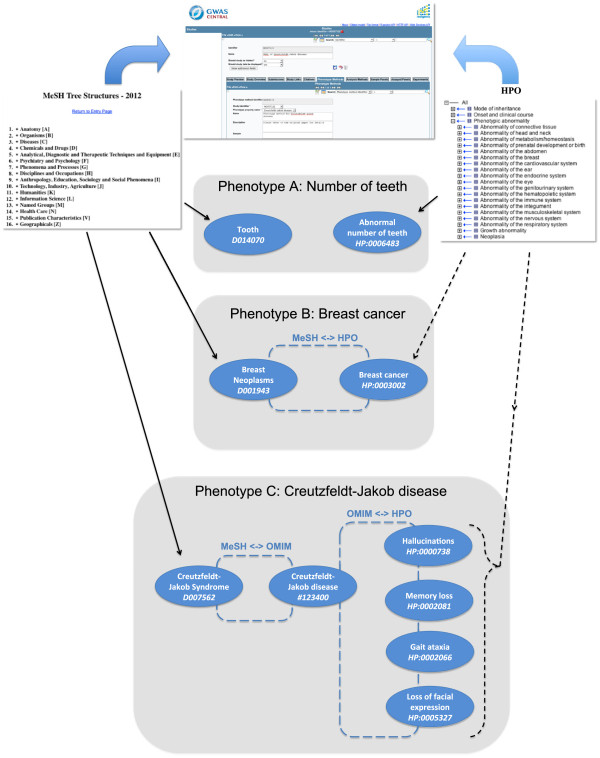
**The phenotype annotation process in GWAS Central as applied to three different phenotypes.** Manual annotations are made using the GWAS Central curation tool. Solid black lines denote direct manual annotations and the dotted black lines denote automatically mapped annotations. ‘Phenotype A’ is manually annotated with a more specific term from HPO. ‘Phenotype B’ is annotated with MeSH and the HPO term is automatically mapped. ‘Phenotype C’ is annotated with a MeSH disease Descriptor and is mapped to the inferred HPO phenotypic abnormalities via OMIM.

In summary, all phenotypes in GWAS Central have a direct MeSH annotation and either a direct HPO annotation, or a mapped HPO annotation, or a mapped set of HPO annotations, describing inferred clinical manifestations, for MeSH disease Descriptors (Figure 4).

### Comparing phenotypes using ontologies: a human-mouse comparative pipeline

The Mammalian Phenotype Ontology (MPO) [46] is used for classifying and organising phenotypic information related to the mouse and other mammalian species. MPO is the *de facto* standard for annotating mouse phenotypes in online resources. As a first step towards high-throughput phenotype comparisons between human and mouse, we have developed an analysis pipeline for the automatic retrieval of human and mouse ontology-annotated phenotype data for gene orthologs. A public version of this pipeline is available from the scientific workflow exchange community website myExperiment [47].

The human-mouse comparative pipeline works as follows:

Starting from a list of human gene symbols, the mouse gene orthologs are determined.

GWAS Central is then queried for phenotypes associated with genes on the list for a given p-value threshold, and the corresponding MeSH annotation(s) retrieved. Each p-value represents the probability of obtaining the observed association between a genetic marker and a phenotype for the dataset, assuming the null hypothesis is true.

Next, the MGD is queried for MPO annotation(s) for the mouse ortholog genes.

Finally, EuroPhenome is queried for MPO annotation(s) made to the mouse orthologs for a given statistical significance limit.

The resulting lists present the ontology annotations made for the gene ortholog dataset and can be used for cross-species comparisons.

The following use case presents an example of the input and output of the pipeline:

The human *BAZ1B* gene is known to be deleted in the development disorder Williams syndrome [48]. A researcher working on *BAZ1B* wishes to learn which phenotypes have been associated with the gene as a result of GWAS, and also which phenotypes have been associated with the mouse ortholog *Baz1b* gene. The researcher downloads the comparative pipeline from myExperiment and loads it into the Taverna workbench [49] installed on their PC.

Before running the pipeline the researcher enters the three required input parameters: the gene “BAZ1B”; the significant GWAS Central p-value threshold of “7” (10e-7); and the EuroPhenome statistical significance limit of “0.00001”. The output includes three annotations from GWAS Central, three annotations from EuroPhenome as a result of the high-throughput phenotyping of a *Baz1b* knockout mouse line, and 28 annotations from MGD derived from published and other sources (Table 2). Manual inspection of these results shows that both GWAS Central and EuroPhenome annotations relate to lipid phenotypes (e.g. the GWAS Central MeSH annotation “Triglycerides” and the EuroPhenome MPO annotations “decreased circulating cholesterol level” and “decreased circulating HDL cholesterol level”).

**Table 2:** Output from running the human-mouse phenotype comparison pipeline

**GWAS Central**	**EuroPhenome**	**MGD**
Plasma	Decreased body weight	Short mandible
Protein C	Decreased circulating cholesterol level	Abnormal myocardial trabeculae morphology
Triglycerides	Decreased circulating HDL cholesterol level	Abnormal myocardium layer morphology
		Malocclusion
		Broad head
		Short snout
		Decreased body size
		Decreased litter size
		Postnatal lethality
		Micrognathia
		Decreased fetal size
		Small parietal bone
		Short nasal bone
		Abnormal palatine bone morphology
		Hypercalcemia
		Abnormal heart morphology
		Double outlet right ventricle
		Dilated heart left ventricle
		Dilated heart right ventricle
		Aorta coarctation
		Abnormal fourth branchial arch morphology
		Decreased birth body size
		Atrial septal defect
		Muscular ventricular septal defect
		Increased heart right ventricle size
		Increased heart left ventricle size
		Abnormal double-strand DNA break repair
		Complete postnatal lethality

The pipeline was used to search for annotations relating to the human *BAZ1B* gene and its mouse ortholog. The human phenotype annotations (GWAS Central) are made using MeSH and the mouse phenotype annotations (EuroPhenome and MGD) are made using MPO. A GWAS Central p-value threshold of “7” (10e-7) and a EuroPhenome statistical significance level of “0.00001” were used. All MGD annotations associated to the mouse *Baz1b* gene were returned.

Follow-up searches of the primary data held in the respective databases are carried out to understand the annotations. GWAS Central shows a genetic marker in the *BAZ1B* gene (SNP rs1178979) with a high probability (p-value 2e-12) of being associated with genetically determining triglycerides, as determined during a GWAS involving white European and Indian Asian participants (see http://www.gwascentral.org/study/HGVST626). EuroPhenome shows that during the “Clinical Chemistry” procedure of a high-throughput phenotyping pipeline [50], the male *Baz1b* heterozygous knockout mouse line was detected as having decreased circulating cholesterol (p-value 7.76e-7) and HDL cholesterol (p-value 8.20e-6) levels compared to the background mouse strains. Taken together, these findings tentatively suggest a role for *BAZ1B* and its ortholog as a genetic determinant of circulating lipids in the human and mouse. The MGD annotations do not include a “lipid-type” phenotype, which may imply that this genotype-phenotype association has not been reported in the literature for the mouse.

Based on the reported association of the *BAZ1B* gene with the circulating lipid phenotype, and knowing that the *Baz1b* knockout mouse line is available (since annotations were obtained from EuroPhenome), the researcher could now prioritise further investigation of the *BAZ1B* gene and its orthologs.

### Genotype to phenotype associations as nanopublications

**Figure 5:**
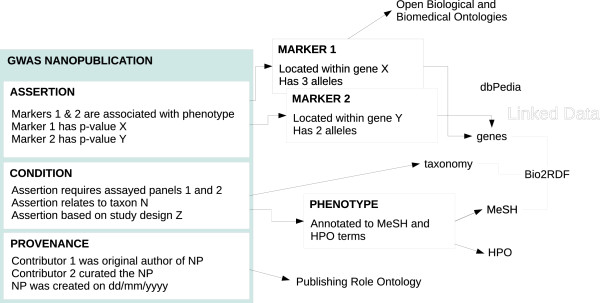
**A schematic representation of GWAS nanopublications and their relationship to the Semantic Web and Linked Data.** Example concepts in the assertion, condition and provenance sections of a nanopublication are shown, along with connections to GWAS Central RDF resources (markers and phenotypes) and external Linked Data resources. Key external resources include MeSH and HPO, scientific articles indexed in PubMed, genes (through Bio2RDF), dbPedia [68], the Ontology for Biomedical Investigations [69] and the Publishing Roles Ontology [70]. RDF data for specific resources is provided via URIs for individual GWAS Central nanopublications, markers and phenotypes. Arrows indicate connections between resources; lines indicate resources are part of a collection (e.g. Bio2RDF). “NP” is used to denote “nanopublication”.

We designed and created nanopublications (following the OpenPHACTS guidelines [51] where possible), related resources, and a query tool for RDF-based GWAS data in GWAS Central. To this end, we attempted to reuse ontologies and to link to existing resources. Figure 5 shows a schematic representation of a GWAS nanopublication and its connection to other external, semantically-enabled, resources. The entire nanopublication dataset, created from the primary GWAS Central relational database, has also been loaded into a triple-store. The triple-store can be queried through the GWAS Central SPARQL end-point. To execute a SPARQL query against the triple-store a researcher can either enter a query in the “GWAS Central SPARQL query form” page (accessible from the start page), or via the API by sending an HTTP GET or POST request containing a ‘query’ parameter to the web service http://fuseki.gwascentral.org/gc/query.

It is important to note that since nanopublications are entirely RDF based and intended for consumption by machines, by themselves they are not human-readable. For user-friendly tools to query and visualise the information contained within GWAS Central, researchers are advised to use the main GWAS Central website [http://www.gwascentral.org].

**Figure 6:**
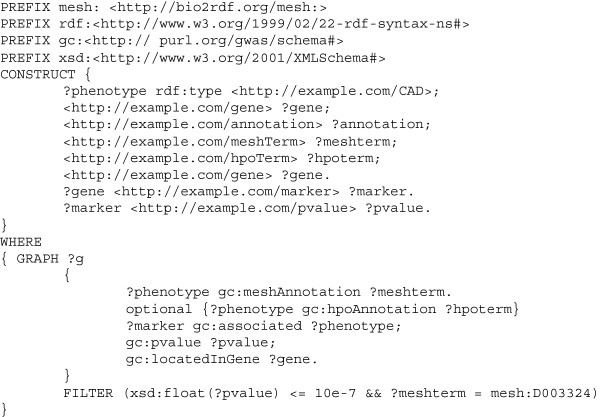
**An example SPARQL query for use case 1.** The SPARQL query run by a researcher who wants to use GWAS Central to obtain an RDF graph of genes, their associated markers and the p-values for all key associations, with a p-value threshold of 10e-7, from nanopublications related to coronary artery disease.

**Figure 7:**
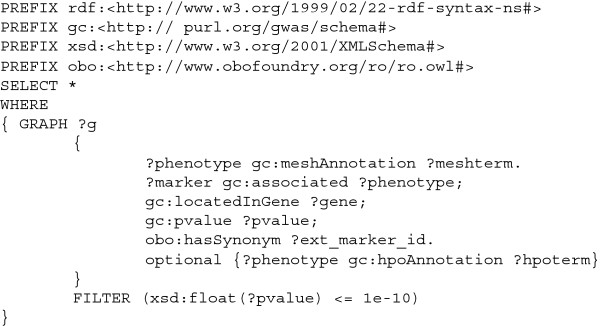
**An example SPARQL query for use case 2.** The SPARQL query run by a researcher who wants to use GWAS Central to retrieve all MeSH and HPO terms and associated information (including external marker IDs) from nanopublications where there are one or more p-values ≤ 10e-10.

With two use cases we can illustrate the application of SPARQL queries against GWAS Central to gain biological insight. Figure 6 shows the SPARQL query used by a researcher who wants to obtain an RDF graph of genes, their associated markers and the p-values for all key associations, with a p-value threshold of 10e-7, from nanopublications related to coronary artery disease (knowing the MeSH Descriptor identifier for coronary artery disease is “D003324”). Figure 7 shows the SPARQL query used by a researcher who wants to retrieve all MeSH and HPO terms and associated information (including external marker IDs) from nanopublications where there are one or more p-values ≤ 10e-10.

Further information on using the Semantic Web resources available through GWAS Central is available from the website help pages [http://www.gwascentral.org/info/web-services/semantic-web-resources].

## Discussion

### Selecting a suitable ontology and annotating phenotypes

We adopted the use of MeSH to define GWAS phenotypes to meet the overriding requirement of being able to capture and organise all data within a single ontology for querying and comparison within GWAS Central. While SNOMED CT scored slightly higher in our automatic annotation analysis compared to MeSH, there are doubts over the suitability of SNOMED CT for use by biomedical researchers. SNOMED CT is a clinical terminology, and has been adopted by the NHS for use as a coding standard. However, concerns have been raised regarding its complexity having a detrimental impact on finding data coded to it [52]. MeSH is more intuitive to biomedical researchers and has been shown to be capable of annotating all GWAS phenotypes at an informative level of granularity, albeit at a coarser granularity than originally described in some cases.

In order to assist our phenotype annotation process we have investigated the use of text-mining and mark-up tools to automate the extraction of relevant phenotype ontology terms from the GWAS literature. We focussed on the annotation of GWAS phenotypes with MeSH, since MeSH forms the “backbone” of GWAS Central annotations. A range of tools are available for the automatic annotation of free-text with MeSH Terms (see [53] for a review of four distinct methods for classifying text with MeSH). We investigated two tools that are well documented and are currently supported: the NCBO Annotator [54] and MetaMap [55]. Both tools were used to annotate a subset of ten full-text GWAS articles with MeSH Terms. Curators also assessed the same subset and assigned MeSH Terms manually following the GWAS Central phenotype annotation process (see Methods).

While a detailed analysis of how the automated tools performed is out of the scope of this article, there was one commonality. Both tools could assign MeSH Terms (including phenotype-relevant terms) to GWAS studies as a whole, however during the manual annotation process MeSH Terms could be assigned to individual GWAS experiments in keeping with the GWAS Central data model. Currently, GWAS Central represents studies that are described in 147 different journal titles, with varying editorial styles. GWAS metadata is complex and understanding the associations between participant panels, methods, observations and genetic marker datasets, as required by the data model, can be challenging for expert curators.

For these reasons, we conclude that there is currently little benefit in incorporating automatic text-annotation using the tools we have evaluated. Nonetheless, we are encouraged to further investigate the possibility of building on the principles of these tools and to develop an advanced text-mining and annotation strategy for future use in GWAS Central.

In the intervening years since the inception of HGVbaseG2P, and subsequently GWAS Central, complementary GWAS databases have embraced the benefits of using controlled vocabularies for the description of phenotypes. Two GWAS databases that currently make use of controlled vocabularies are the DistiLD database [56] and GWASdb [57].

The DistiLD database (reported in 2011) maps GWAS SNPs to linkage disequilibrium blocks and diseases where ICD10 is used to define the diseases. ICD10 is an ideal vocabulary for the description of disease phenotypes, but, as expected, resolution is lost when querying the dataset for non-disease traits. For example, a search for “blood pressure” on the main search page [http://distild.jensenlab.org] simply returns results from free-text searches of the publication titles and abstracts.

GWASdb (reported in 2011) allows exploration of genetic variants and their functional inferences, incorporating data from other databases including GWAS Central. Seventy percent of phenotypes in GWASdb are mapped to DOLite and the remainder are mapped to HPO [57]. This prevents the use of a single ontology to query against the complete dataset. It is also unclear from the interface as to the level of granularity of the annotations, with only the first four levels of HPO accessible from the browser. By contrast, GWAS Central annotates up to level nine of HPO and it is therefore difficult to assess whether GWAS Central and GWASdb annotations agree for a given study.

A wider question remains as to the reproducibility of phenotype annotations between databases and the interchange of data bound to different standards. We have initiated coordination between complementary GWAS databases to ensure a unified set of annotations exist, mapped to all relevant semantic standards in use in the community (see the “GWAS PhenoMap” project at http://www.gwascentral.org/gwasphenomap/).

### Cross-species phenotype analysis

Our human-mouse phenotype comparison pipeline facilitates immediate retrieval of ontology-bound phenotype data for orthologous genes. Orthologous genes that do not share a phenotype could be novel candidates for the phenotype and thus could benefit from undergoing further study.

Phenotypes can be logically defined using ontologies by making an equivalence between terms in a pre-composed ontology (e.g. MeSH, HPO and MPO) and *entity* and *quality* (EQ) decompositions [26]. For example, the MPO term “supernumerary teeth” is represented in EQ as “*E: tooth + Q: having extra physical parts*” (taken from the OBO Foundry mammalian phenotype logical definitions).

Comparison of the phenotypes generated from our pipeline is currently a manual process, but this could be optimised through using the EQ logical definitions of the pre-composed ontology terms. This would provide computer-interpretable definitions that could support reasoning to suggest, for example, that the MPO term “supernumerary teeth” and the HPO term “Increased number of teeth”, represented by the same logical definition (using a species-neutral anatomy ontology), are equivalent.

Encouragingly, work has begun on decomposing HPO musculoskeletal related terms into EQ definitions for the purpose of cross-species comparisons [44]. As the EQ definition layer is progressed by domain experts into other categories of phenotypes covered by HPO, the possibility of making GWAS phenotypes available as EQ statements advances closer.

In an alternative approach, the PhenoHM human-mouse phenotype comparison server accepts phenotypes as input, rather than genes, and implements direct mappings from human (HPO) to mouse (MPO) ontologies [58] to identify human and mouse genes with conserved phenotypes. By comparison, our pipeline provides the flexibility to allow phenotypes from any ontology to be manually compared (from any database providing the relevant web services) and in theory the PhenoHM mappings could be extended to include MeSH and other ontologies. However, evaluation is required of the benefits of producing relatively quick *ad hoc* mappings between terminologies compared to a more time-consuming logical definition process that could facilitate more extensive cross-ontology comparisons.

Whichever method is employed, it will make reversing the pipeline an attractive possibility. Lists of orthologous phenotypes could serve as input for querying against human and mouse resources to retrieve associated genes, in order to answer questions such as “which gene is responsible for this phenotype in the mouse?”. In the immediate term we anticipate that the rich, high-quality GWAS phenotype annotations in GWAS Central will enhance the results of current and future cross-species comparisons involving the human.

### Semantic GWAS data nanopublishing

By making genotype-phenotype associations available in a Linked Data-friendly form [59], GWAS Central has taken the first steps towards interoperability on the Semantic Web. Our prototype nanopublications were designed to link with and mesh into the broader web of Linked Data, by way of shared URI identifiers and ontologies for identifying and describing key entities in our domain of interest. This first-generation collection of GWAS nanopublications, though limited in scope and features, holds great potential for enriching the expanding network of semantically-enabled online information resources in the biomedical sphere.

It is important to emphasise that GWAS Central nanopublications are simply items of data, not statements of knowledge. For example, a p-value for a marker in a GWAS represents a statistical test of association that was factually observed in an experiment. This p-value is clearly not equivalent to a validated biological causal relationship between a genetic variant and a disease. There is some risk that eventual users of the data may confuse the two, especially given that GWAS nanopublications will be distributed widely and consumed outside of the “parent” GWAS Central resource itself. This is not a reason to avoid nanopublishing as such, but it does underline the importance of including appropriate metadata describing context and provenance along with, and clearly linked to, the core assertions.

As new tools are developed to reduce the technical knowledge required to semantically enable resources (e.g. the D2RQ Platform [60] and Triplify [61]) and leave bioinformaticians with the job of simply organising their data, it seems obvious that increasing numbers of biomedical resources will become semantically enabled in the near future. As and when this happens, we intend to further expand the set of Linked Data resources that our GWAS nanopublications link out to, thereby increasing their utility when consumed by other semantic tools. We are also planning to further expand the semantic capabilities of GWAS Central by exposing the association nanopublications, the SPARQL endpoint and the phenotype comparison pipeline (and future workflows we may develop) via the SADI framework.

## Conclusions

We have made available high-quality phenotype annotations within a comprehensive GWAS database. We have considered the spectrum of phenotypes reported by published GWAS, ranging from diseases and syndromes to individual medical signs and symptoms, and adopted a suitable annotation framework to capture phenotypes at the finest level of granularity. All GWAS phenotypes are bound to a MeSH Descriptor to ensure the pragmatic necessity that a single ontology can be queried to retrieve all phenotype data. The HPO provides single phenotypic abnormality annotations either directly, mapped from MeSH, or inferred via deconstructions of disease phenotypes. A human-mouse phenotype comparative pipeline provides a valuable tool for comparison of human and mouse phenotypes for orthologous genes.

By providing GWAS Central data in the form of nanopublications and integrating this data into the Linked Data web, we present a platform from which interesting and serendipitous findings related to genotypes, phenotypes, and potentially other types of Linked Data, can be made.

## Methods

### Analysis of ontologies for describing GWAS phenotypes

In order to assess ontology suitability (defined as “the ability to capture the maximum number of phenotypes at the level of granularity at which they are described”), we compared our phenotype/trait descriptions against terms in BioPortal. Initially, we exported the 1046 unique ‘phenotype’ free-text descriptions obtained from the published GWAS reports and other external sources to a tab-separated file, resulting in a text list of phenotypes. Before the list was compared against ontologies, the text was made consistent (normalised) through a combination of manual and automated steps:

1. In a manual step all descriptions were assessed to determine if they related to a trait or phenotype. To ensure consistency in the descriptions, and since the majority of descriptions related to traits, phenotypes were transformed to traits. This involved the removal of values assigned to traits e.g. “Hair color: black versus red” was transformed to the trait “Hair color”.

2. Since the ontologies under investigation express concepts in the singular form, we ran a script to remove plurals from the trait list.

3. British and American spellings are not synonymous in all ontologies, for example the HPO term “Abnormality of the esophagus” (HP:0002031) does not have the synonym “Abnormality of the oesophagus”. Therefore, British and American spelling differences were neutralised by providing both spellings for a word. A script split each trait description (term) into component strings (words) and queried the words against a list of words with spelling variants (source: http://en.wikipedia.org/wiki/Wikipedia:List_of_spelling_variants). Where a word was found to have a spelling variant a new term was created containing the word with the alternative spelling. The new term was appended, tab-separated, to the original term in the trait list.

The BioPortal REST web services allow for programmatic querying and comparison of the ontologies contained within BioPortal. In order to access the web services users are required to login to BioPortal to obtain an API key. The ‘Search’ web service queries a user-specified term against the latest versions of all BioPortal ontologies, thus eliminating the need to parse the latest version of an ontology in its native file format (e.g. OWL, OBO, UMLS format or custom XML). The ‘Search’ web service ignores capitalisation of both the user-specified term and the ontology terms. By default, the search attempts to find both partial and exact matches. During a partial search for a single word the wildcard character (*) is automatically appended to the end of the word, and for multiple-word searches the wildcard character is appended to the end of each word [62]. The next stage of our analysis involved running a script to query each trait description against all BioPortal ontologies using the ‘Search’ web service. The web service was run twice for each term, with alternating ‘exact match’ arguments – this argument forces an exact match. During both runs for each trait description, the input was the normalised term, for example “Hair color”. The web service output was queried for matches in the ontologies of interest, namely DO, HPO, ICD10, MeSH and SNOMED CT. If a spelling variant did not return a match in at least one of the ontologies of interest, then the spelling alternative was also queried, for example “Hair colour”. The query term and the mapped ontology term were written to an output file. The total numbers of trait descriptions that map exactly and partially to the ontologies under investigation were recorded (Table 1). When a trait was mapped to a single term in only one of the ontologies (a unique mapping), the query term, the mapped ontology term and the ontology name were written to a second output file. The number of unique mappings for each ontology during the exact and partial searches was recorded (Table 1).

### Ontology annotation and mapping

The initial ontology association between a phenotype and a genetic marker dataset is made during a manual curation process with the subsequent mappings made automatically. We use the MOLGENIS database management platform [63] as the basis for a curation tool. The GWAS Central data model can be viewed and edited through a series of connected forms (Figure 4). For each GWAS represented in GWAS Central a curator obtains the full-text report for the study and adds a new “sub-study” for each experiment. As the information is obtained from reading the report, the metadata for each experiment is entered into the curation tool to satisfy the GWAS Central data model, resulting in an experiment that is associated with sample panels, phenotype methods, analysis methods and a genetic marker dataset (see the GWAS Central glossary: http://www.gwascentral.org/info/reference/definitions-and-glossary). Each phenotype method contains a phenotype property that requires a phenotype annotation. The relevant MeSH Descriptor identifier is entered into the form. If a curator deems the annotation not to be an exact match, and instead the annotation is made using the closest available term, then this is flagged in the database. In these cases an appropriate HPO term will be manually sought.

MeSH is automatically mapped to HPO via UMLS. The cross-referenced UMLS concept unique identifier for a HPO term is obtained either from the source HPO OBO file http://compbio.charite.de/svn/hpo/trunk/src/ontology/human-phenotype-ontology.obo or via MetaMap [55], which maps free-text to the UMLS Metathesaurus. The MeSH identifier is then obtained from the cross-referenced UMLS entry. The HPO-to-OMIM mappings are automatically extracted from the mapping file downloaded from the HPO group’s website http://compbio.charite.de/svn/hpo/trunk/src/annotation/. The OMIM-to-MeSH mappings are manually assigned.

### Phenotype comparison pipeline

The human-mouse phenotype comparison pipeline uses the web services made available by the contributing data sources to ensure the latest data is accessed. A number of web services were used to return mouse ortholog genes for a list of human gene symbols and then return the corresponding annotated phenotypes for both sets. The Entrez Programming Utilities (E-Utilities) ESearch service [64] is used to validate the given list and retrieve Entrez IDs for the genes. The gene symbols for the mouse orthologs are retrieved from the MGI BioMart [65]. The MGI and EuroPhenome BioMarts are accessed to retrieve the MPO terms annotated to the mouse ortholog gene list. The GWAS Central REST web service is accessed to retrieve the phenotype annotations for the human gene list. The public version of the pipeline was created using the workflow management system Taverna [49]. Taverna offers users the ability to visualise and reuse web services within workflows via the Taverna workbench, which is an intuitive desktop client application. Taverna is also integrated with myExperiment, so facilitating the distribution of the pipeline and its reuse by the community in whole or in part.

### RDF and nanopublications

To provide semantically enabled GWAS Central resources and integrate them into the Linked Data web, Perl modules originally created to search markers, phenotypes, association results and nanopublications in GWAS Central were extended to provide output in RDF, Turtle and in the case of nanopublications, N-Quads format. When navigating resources, the format to be returned to client applications is determined either through HTTP header content-type negotiation (application/rdf + xml, text/turtle or text/x-nquads), or through the use of a 'format' parameter (rdfxml, turtle or nquads) in the URI.

A Perl script utilising the above-mentioned search modules extracted all appropriate resources from GWAS Central as RDF, which were subsequently loaded into an RDF triple-store created using the Apache Jena TDB component [66]. Jena was selected due to its support for the named graph extension that is an essential requirement for representing individual sections within nanopublications. The SPARQL end-point was set up using the Fuseki server [67].

Using the methodology of other GWAS data resources [4], we deem results with a p-value less than 10e-5 as showing an association and so these are included in our nanopublications. An example GWAS nanopublication and its associated connections with key external resources [68-70] are shown in Figure 5.

## Availability of supporting data

The GWAS Central phenotype annotations can be queried and viewed from the web interface at: http://www.gwascentral.org/phenotypes.

The GWAS Central SPARQL end-point can be accessed at: http://fuseki.gwascentral.org.

The human-mouse comparative phenotype pipeline described in this paper, named “get human and mouse phenotypes for a gene”, is available from myExperiment at: http://www.myexperiment.org/workflows/2131.html.

## Abbreviations

DO: Disease Ontology; GWAS: Genome-wide association study/studies; HPO: Human Phenotype Ontology; ICD: International Classification of Diseases; MeSH: Medical Subject Headings; MGD: Mouse Genome Database; MPO: Mammalian Phenotype Ontology; OBO: Open Biological and Biomedical Ontologies; OMIM: Online Mendelian Inheritance in Man; RDF: Resource Description Framework; SNOMED CT: Systematized Nomenclature of Medicine - Clinical Terms; UMLS: Unified Medical Language System.

## Competing interests

The authors declare that they have no competing interests.

## Authors' contributions

TB conceived of the study, carried out the research and drafted the manuscript. RCF conceived of the RDF components and implemented them. GAT conceived of the study and edited the manuscript. AJB conceived of the study and supervised all aspects of the research. All authors read and approved the final manuscript.
